# The Controlling Nutritional Status (CONUT) Score for Prediction of Microvascular Flap Complications in Reconstructive Surgery

**DOI:** 10.3390/jcm12144794

**Published:** 2023-07-20

**Authors:** Rihards P. Rocans, Janis Zarins, Evita Bine, Renars Deksnis, Margarita Citovica, Simona Donina, Biruta Mamaja

**Affiliations:** 1 Intensive Care Clinic, Riga East Clinical University Hospital, Hipokrata Street 2, LV-1079 Riga, Latvia; evitabine@gmail.com; 2 Department of Anaesthesia and Intensive Care, Riga Stradins University, Dzirciema Street 16, LV-1007 Riga, Latvia; biruta.mamaja@aslimnica.lv; 3 Department of Hand and Plastic Surgery, Microsurgery Centre of Latvia, Brivibas Street 410, LV-1024 Riga, Latvia; janis.zarins@mcl.lv; 4 Baltic Biomaterials Centre of Excellence, Headquarters at Riga Technical University, Pulka Street 3, LV-1007 Riga, Latvia; 5 Surgical Oncology Clinic, Riga East Clinical University Hospital, Hipokrata Street 4, LV-1079 Riga, Latvia; renars.deksnis@gmail.com; 6 Laboratory Department, Riga East Clinical University Hospital, Hipokrata Street 2, LV-1079 Riga, Latvia; margarita.citovica@aslimnica.lv; 7 Institute of Microbiology and Virology, Riga Stradins University, Ratsupites Street 5, LV-1067 Riga, Latvia; donsimon@inbox.lv

**Keywords:** controlling nutritional status, microvascular flap complications, reconstructive surgery

## Abstract

Microvascular flap surgery is a widely acknowledged procedure for significant defect reconstruction. Multiple flap complication risk factors have been identified, yet there are limited data on laboratory biomarkers for the prediction of flap loss. The controlling nutritional status (CONUT) score has demonstrated good postoperative outcome assessment ability in diverse surgical populations. We aim to assess the predictive value of the CONUT score for complications in microvascular flap surgery. This prospective cohort study includes 72 adult patients undergoing elective microvascular flap surgery. Preoperative blood draws for analysis of full blood count, total plasma cholesterol, and albumin concentrations were collected on the day of surgery before crystalloid infusion. Postoperative data on flap complications and duration of hospitalization were obtained. The overall complication rate was 15.2%. True flap loss with vascular compromise occurred in 5.6%. No differences in flap complications were found between different areas of reconstruction, anatomical flap types, or indications for surgery. Obesity was more common in patients with flap complications (*p* = 0.01). The CONUT score had an AUC of 0.813 (0.659–0.967, *p* = 0.012) for predicting complications other than true flap loss due to vascular compromise. A CONUT score > 2 was indicated as optimal during cut-off analysis (*p* = 0.022). Patients with flap complications had a longer duration of hospitalization (13.55, 10.99–16.11 vs. 25.38, 14.82–35.93; *p* = 0.004). Our findings indicate that the CONUT score has considerable predictive value in microvascular flap surgery.

## 1. Introduction

Microvascular flap surgery has become a generally acknowledged procedure for significant defect reconstruction. Complex microvascular techniques and in-depth knowledge of blood rheology and microanastomosis function are required for this kind of surgery. Although substantial progress has been achieved in preventing complications, the rate of flap loss is still significant (1–7.1%) and can have significant adverse effects on the patient [1,2]. Flap thrombosis, flap hematoma, and flap loss are the most frequent and severe major surgical complications [3]. Mechanical problems comprise the most frequent causes of late flap failure (>48 h), and impaired arterial and venous blood supply is the most widespread cause of early flap failure (<48 h) [4]. Problematic and delayed healing, wound dehiscence, infection, fistula, and donor site problems are considered minor surgical complications. Even though microvascular flap transplantation relies on greatly specific surgical concepts, the issue of systemic reaction to surgical trauma and tissue healing is just as relevant here as in other types of surgery [5]. 

The most common indications for microvascular flap surgery are primary oncology or trauma, as well as defects related to previous surgery or infection [1]. Malnutrition may be common in patients requiring microvascular flap surgery [6], as many indications for microvascular flap surgery are also risk factors for poor nutritional status [7]. Previous studies show that the presence of malnutrition is a considerable risk factor for surgical complications in different patient populations [6,7,8,9,10]. Malnourished patients are at a higher risk of surgical complications such as wound dehiscence, infection, and fistula formation [9,11]. Most of these complications require reoperation, which can further increase patient morbidity and hospital costs [12]. Screening, assessing, and managing these patients is important because malnutrition is a modifiable pre-operative risk factor that, if addressed early, can reduce the risk of post-operative complications [13]. Given the complexity of microvascular flap transplantation and the availability of nutritional treatment strategies, a systematic approach to addressing nutrition risk significantly improves surgical outcomes in microvascular flap surgery [6,7].

The objective measurement of nutritional status can be performed with a wide range of tools, although there is no “gold standard” approach for measuring malnutrition [14]. The use of laboratory biomarkers for screening and assessing nutrition risk may be convenient, since laboratory evaluation is already routinely performed for preoperative assessment. Multiple studies have elucidated the link between laboratory biomarkers of poor nutritional status and surgical complications [6,7,8]. Studies have shown that lymphocyte count, albumin, prealbumin, and total plasma cholesterol are markers for poor nutritional status and can be quantified using nutritional assessment tools [15,16]. The controlling nutritional status (CONUT) score is an evolving tool that has demonstrated good postoperative outcome assessment ability in diverse surgical populations [9,17]. It is intended for inpatient assessment and is relatively simple to use, as it is calculated using only three values: serum albumin level, total cholesterol level, and total lymphocyte count [16]. A CONUT score of 0–1 is defined as no nutrition risk, and higher scores are defined as higher degrees of nutrition risk [16]. CONUT could be applied for assessment of nutrition risk in microvascular flap surgery due to its broad applicability and previous evidence for predicting complications in various surgical populations. The purpose of this study is to assess the predictive value of the CONUT score for predicting complications in elective microvascular flap surgery.

## 2. Materials and Methods

The study protocol and the informed consent form were approved by the Ethics Committee of Riga Stradins University (Approval Number 22-2/399/2021), and by the Science Department of Riga East University hospital (Approval Number Nr.AP/08-08/22/135).

### 2.1. Patient Selection

This prospective cohort study included 72 patients undergoing elective microvascular flap transplantation surgery at Riga East University Hospital from the 1 October 2021 to the 31 January 2023. Given the observational nature of our study, all surgical, anesthesia, and clinical management decisions were made by the attending physicians. The inclusion criterion was adult patients undergoing elective microvascular flap transplantation. The exclusion criteria were patients with sepsis or severe systemic bacterial infection; patients with autoimmune disorders; patients with blood-borne viral infections (Hepatitis B; Hepatitis C and HIV); pregnant patients and patients during lactation period; and patients with congenital hypercoagulability or any clotting disorder. 

### 2.2. Anaesthesia and Surgical Protocol

All patients received general anesthesia (GA). Starting at the induction of anesthesia electrocardiography, pulse oximetry, noninvasive blood pressure, and end-tidal carbon dioxide concentration were monitored in all patients. Induction was performed using fentanyl (Fentanyl-Kalceks^®^ 0.05 mg/mL, A/S Kalceks, Riga, Latvia) 1.5–2 μg/kg, and propofol (Propofol^®^ 10 mg/mL, Fresenius Kabi AG, Bad Homburg, Germany) 1–2 mg/kg intravenously (iv). GA was maintained using sevoflurane (Sevorane^®^, AbbVie S.r.l., Campoverde, Italy) 0.8–1.2 MAC, and continuous analgesia was provided with fentanyl 1–1.5 μg/kg/h. Cisatracurium (Nimbex 2 mg/mL, Aspen Pharma Ltd., Dublin, Ireland) 0.15 mg/kg iv was used for tracheal intubation, followed by a continuous infusion of 1–2 μg/kg/min for muscle relaxation. Crystalloid infusion (RiLac, B. Braun Melsungen AG, Melsungen, Germany) was administered at a rate of 3.5 to 6.0 mL/kg iv per hour during surgery and the early postoperative period, with a target urine output of 1–2 mL/kg/h. Colloid fluid (Gelofusine, B. Braun Melsungen AG, Melsungen, Germany) was administered when an estimated blood loss of >500 mL occurred during surgery. Patients received both peripheral and central temperature monitoring during surgery to avoid hypothermia. Patients were administered vasopressors, such as ephedrine (Ephedrine Sintetica, Sintetica GmbH, Münster, Germany) or norepinephrine (Norepinephrine Sopharma, Sopharma AD, Sofia, Bulgaria), when their mean arterial blood pressure was below 65 mmHg for more than 5 min. Peripheral nerve blocks with ultrasound and neurostimulation guidance were performed when indicated. Patients received close postoperative monitoring of vital signs, fluid balance, and postoperative pain management in the post-anesthesia care unit. Postoperative thromboprophylaxis was provided with enoxaparin (Clexane^®^, Sanofi-Aventis S.A., Barcelona, Spain) 40 mg once daily from the first postoperative day for all patients. During and after surgery, patients with clinical symptoms of excessive blood loss or those with hemoglobin < 7 g/dL received blood product transfusions. All operations were performed by a team of highly experienced surgeons. The selection of flap type was based on the tissue type necessary for defect site reconstruction, the size of defect, the length of the pedicle, and the patient’s positioning during surgery. The flaps used in the study were the anterolateral thigh flap, deep inferior epigastric artery perforator flap, fibular flap; radial free forearm flap, gracilis muscle flap, temporal artery flap, serratus anterior flap, latissimus dorsi flap, and medial condyle flap. The team of surgeons closely monitored the microvascular flap for the first five postoperative days. Flap patency was assessed using clinical assessment of flap color, temperature, tissue turgor, and capillary refill. 

### 2.3. Data Collection

Blood draws were obtained on the day of surgery immediately upon the first arrival in the operating room before initiation of the first crystalloid infusion. Full blood count analysis was performed using the XN-1000 system (Sysmex Europe SE, Norderstedt, Germany). Concentrations of albumin were analyzed using the colorimetric method (Cobas C, Roche, Manheim, Germany). Concentrations of total plasma cholesterol were analyzed using the Enzymatic colorimetric method (Cobas C, Roche, Manheim, Germany). The serum albumin concentration, total peripheral lymphocyte count, and serum total cholesterol concentration were used to assign the CONUT score. As seen in Table 1, the CONUT score was determined by assigning laboratory values according to the tool first used by Ignacio de Ulíbarri and coauthors [16].

Demographic data, comorbidities, data on perioperative course, anesthesia care, surgical outcome, length of stay in the intensive care unit (ICU), and total duration of hospitalization were obtained from written and electronic health records according to a previously defined protocol. Patients received postoperative daily follow up until discharge from the hospital.

### 2.4. Definitions

True flap loss was defined as flap blood supply deficiency due to arterial or venous anastomosis dysfunction or thrombosis that leads to complete loss of the transplanted flap. Other flap complications were defined as any of the following: hematoma (without interfering with flap blood supply), flap wound infection, secondary or incomplete flap wound healing, and partial flap loss. Partial flap loss was defined as the presence of distal marginal flap necrosis with no anastomosis dysfunction. Any flap complication was defined as the presence of either true flap loss or any other flap complication. ICU length of stay was the timing between admission to the ICU and discharge from the ICU to the ward. Hospital length of stay was the timing between admission to the hospital and discharge from the hospital.

### 2.5. Statistical Analysis

Statistical analysis was performed using SPSS Statistics for Windows, Version 26.0. (IBM Corp. Armonk, NY, USA). The Kolmogorov–Smirnov test was used to evaluate whether the datasets conformed to a normal distribution. Continuous variables conforming to normal distribution were presented as mean and CI95, while categorical variables were presented as median ± interquartile range (IQR). Differences in data distribution between the groups were evaluated using the Mann–Whitney U test for non-parametric datasets and the two-sample t-test or ANOVA for datasets conforming with normal distribution. A Chi-square test was applied for nominal variable sets. Binary logistic regression models were used to obtain odds ratios for specific variables. The receiver operator curve (ROC) and area under curve (AUC) were used for evaluating the diagnostic ability of a binary classifier system. Youden’s Index (YI) and the Concordance Probability Method (CZ) was used for defining optimal cut-off values [18]. Statistical significance was assumed if two-tailed *p* < 0.05.

## 3. Results

In total, 72 patients—40 (55.6%) men and 32 (44.4%) women—were included. The mean age was 55.3 years (95% CI95 51.5–59.1). The overall complication rate was 15.2% (*n* = 11). True flap loss with vascular compromise occurred in 5.6% (*n* = 4), with two of these cases being late flap loss (>72 h). Both cases of early true flap loss underwent urgent anastomosis revision. Both cases of late flap loss underwent repeated elective microvascular flap transplantation. Other flap complications occurred in seven cases, with difficult flap healing or partial flap loss occurring in 5.6% (*n* = 4), flap infection occurring in one, and hematoma occurring in two cases. The median number of revisions in patients with true flap loss was 1.5 (IQR 1). The median number of revisions in patients with other flap complications was 1 (IQR 0.75, *p* = 0.223).

As seen in Table 2, there were no significant differences in age or gender distribution in patients with any flap complications or flap loss, and in patients without complications. No significant differences in true flap failure or other flap complications were found between different areas of reconstruction and different anatomical flap types. No significant differences in true flap failure or other flap complications were found between different indications for reconstruction. Of the included comorbidities, obesity was found to be more common in patients with any flap complications (*p* = 0.01). Only two patients had a BMI < 20 kg/m^2^, and there was no statistically significant link between decreased BMI and any flap complications. No statistically significant link was found between BMI and CONUT score. No significant differences in the rates of true flap failure or other flap complications were found in patients with other comorbidities.

As seen in Table 3, no significant links were found between the duration of surgery and anesthesia factors and any flap complications. A higher intraoperative hematocrit was associated with flap complications, with the highest intraoperative hematocrit found in cases with subsequent true flap loss (*p* = 0.009). Only one patient received intraoperative hemotransfusion, and five patients received hemotransfusion in the early postoperative period. There was no significant link between the presence of hemotransfusion and any flap complications.

As seen in Table 4, patients with any flap complications had a significantly lower plasma lymphocyte count (*p* = 0.001). Multivariate regression analysis revealed that an increase in lymphocyte count decreases the incidence of all complications (OR 0.998 CI95 0.996–0.999). Patients with any flap complications had a significantly lower plasma monocyte count (*p* = 0.021). No differences in plasma lymphocyte/monocyte ratio, plasma albumin, and total plasma cholesterol were found in patients with any flap complications.

**Figure 1:**
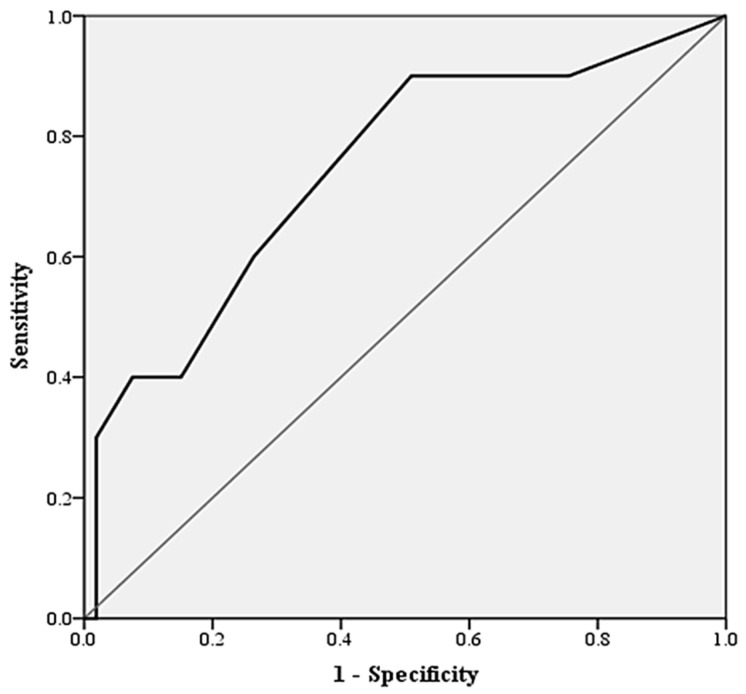
ROC curve characteristics of CONUT score for predicting complications in microvascular flap surgery; receiver operator curve characteristics and area under curve of CONUT score for predicting the presence of flap complications other than true flap loss. CONUT scores had an AUC of 0.813 (CI95 0.659–0.967, *p* = 0.012).

As seen in Figure 1, analysis on the predictive accuracy of CONUT score of other surgical complications found that CONUT score had an AUC of 0.813 (0.659–0.967, *p* = 0.012). A CONUT score of >2 was found to be optimal during cut-off analysis (Sensitivity 21.1%, Specificity 95.6%, PPV 66.7%, NPV 74.1%, *p* = 0.022). CONUT score of >2 increases the odds of other flap complications (OR 5.4, CI95 1.38–20.90, *p* = 0.015). Univariate regression revealed that any increase in CONUT score increased the odds of other flap complications (OR 1.43 1.09–1.85). Patients with any flap complications had a longer duration of hospitalization (13.55, 10.99–16.11 vs. 25.38, 14.82–35.93; *p* = 0.004). There was no difference in duration of ICU stay between patients with flap complications and patients with no flap complications (1.13, 0.03–2.26 vs. 1.50 1.00–2.00, *p* = 0.471).

## 4. Discussion

The main findings of the present study were that an increase in the preoperative CONUT index is a reliable predictor for flap complications, with a CONUT score of >2 being the optimal cut-off for predicting complication risk. Flap complications were found to be linked to lymphocytopenia, monocytopenia, hematocrit, and obesity. The incidence of true flap loss was 6.2%, and the incidence of other less severe complications was 9.2%. The duration of hospitalization was significantly longer in patients who had flap complications. 

Microvascular flap transplantation requires complex microvascular techniques, and flap success relies on the function of microanastomosis and adequate flap perfusion [4]. While these are greatly specific concepts, the issue of systemic reaction to surgical trauma and tissue healing and nutrition is just as relevant here as in other types of surgery [5]. Malnourished patients are more likely to experience complications during and after surgery, longer hospital stays, and a slower recovery time both in the general surgical population [8,9,10] and in microvascular flap surgery [6,7]. Given the complexity of the procedure and severity of the complications, clinical prediction tools regarding nutrition risk may be used during preoperative assessments to identify patients who may require more extensive evaluation or preparation before surgery [6,7]. A study by Yu and co-authors suggests that the prognostic nutritional index (PNI), a score including some of the same parameters as CONUT, can be simply and effectively used to predict free flap failure in extremity reconstruction [6]. Our results indicate that an increased CONUT score significantly increases the odds of postoperative complications. To the best of our knowledge, no previous studies have elucidated the predictive value of CONUT in microvascular flap surgery. However, our findings coincide with data from different surgical populations wherein CONUT has been shown to reliably predict complications and mortality [8,19]. Additionally, our results suggest that patients with flap complications had longer hospital stays. This coincides with previous studies that report longer hospital stays and increased hospital costs in patients who experience free flap failure in breast and head and neck reconstruction [20,21]. Considering that any increase in the CONUT score increases the risk of flap complications it can also consequently lead to longer hospital stays and increased costs.

In our study, we found CONUT > 2 to be the most optimal cut-off value, which also coincides with some data from previous studies in other surgical populations [9,17,22]. It must be noted that we found a CONUT > 2 cut-off value to have a relatively low sensitivity (21.1%) and a high specificity (95.6%). These results imply that a cut-off value of CONUT > 2 is best utilized for excluding patients who are at a low nutrition risk and low risk of subsequent flap complications.

Interestingly, while our data showed CONUT to be a reliable predictor for flap complications, it was not a reliable predictor specifically for true flap loss. This indicates that the pathophysiology of true flap loss due to anastomosis compromise [23] may be separate from the pathophysiology of other surgical complications in microvascular flap surgery. Most minor complications in microvascular flap surgery, such as wound dehiscence, infection, and fistula formation, occur due to inadequate tissue healing and regeneration [24]. These complications may be linked to undernutrition [7] instead of being a direct result of early anastomosis compromise. Notably, even minor complications place the patient at an increased risk of re-exploration or repeated microvascular flap transplantation [25]. Furthermore, patients receiving microvascular flap transplantation are predisposed to difficult wound healing, both at the site of reconstruction and at the donor site [25]. 

Plasma lymphocyte count is a component of CONUT that may have a substantial role in the pathophysiology of microvascular flap complications. Studies in various surgical populations show that patients with preoperative lymphocytopenia had a significantly higher incidence of complications compared to those with a normal lymphocyte level at admission [5,26,27]. Lymphocyte recovery in the first postoperative days could play an important role in the mechanisms of tissue repair, and a primary role in wound healing [28]. Monocytes are the most responsive leukocytes in response to trauma [29] and multiple monocyte immunophenotypic alterations are observed upon surgery [30,31]. In contrast to our findings, Kosec and co-authors did not find a link between preoperative monocyte count and postoperative complications in microvascular flap surgery [5]. 

Multiple patient-related risk factors, including coronary artery disease, diabetes, smoking, peripheral arterial vascular disease, arterial hypertension, and higher ASA score, are related to flap failure [1]. Obesity has been deemed to be a risk factor for poor surgical outcomes in medical care, but the majority of published studies in various surgical populations have been uncertain [32,33,34]. Some previous studies found obesity to be associated with increased perioperative risk in free abdominally based autologous breast reconstruction, which coincides with our findings [35,36]. Conversely, multiple studies have also evidenced that obesity does not increase the risk of postoperative complications in microvascular flap surgery [37,38,39]. However, it must be noted that the presence of obesity does not exclude the presence of double-burden malnutrition, which can also have detrimental effects on overall health [40,41]. Furthermore, the study by Ignacio de Ulíbarri and coauthors found no relationship between BMI and undernutrition in their study population, as BMI is not a reliable indicator for acute malnutrition [16]. Our data indicate that both obesity and nutrition risk increase the rate of flap complications, which indicates that both conditions should be assessed and treated to improve outcomes in microvascular flap surgery.

This study had several limitations. Firstly, given the observational nature of our study, individual surgical, anesthesia, and nutritional management decision-making was performed by the clinicians, and may have varied between cases. Secondly, ours was a single-center study, which affects the possible generalizability of the findings. Notably, a considerable part of our study population has oncology as a primary diagnosis, which likely introduces additional confounding risk factors for surgical complications. Conversely, it must be noted that patients with oncology as a primary diagnosis are very likely to benefit from an assessment of nutrition risk [8,9,15,17]. It should be noted that the presence of radiotherapy, which can present confounding factors, was not considered in this study. Finally, it is important to note that serum albumin, which is an important item in both the CONUT and PNI scores, is not a part of current definitions of malnutrition [42]. Therefore, CONUT score results are considered to be indicators of nutrition risk rather than an assessment of nutritional status. Further studies are needed to clarify the use of nutrition risk assessment tools to predict complications in different patient populations, and to specify the use of specific nutritional interventions to improve outcomes in microvascular flap surgery.

## 5. Conclusions

Assessment of nutritional risk to estimate the risk of microvascular flap complications using the CONUT score has considerable predictive value. Patients undergoing this type of surgery can be evaluated in terms of predicting nutritional risk to optimize decision-making in perioperative care.

## Figures and Tables

**Table 1:** The evaluation of the controlling nutritional status (CONUT) score; the controlling nutritional status (CONUT) score tool as first described by Ignacio de Ullibarri and coauthors [16].

Variable	Undernutrition Degree			
	Normal	Mild	Moderate	Severe
Serum albumin (g/dL)	≥3.50	3.00–3.49	2.50–2.99	<2.50
Score	0	2	4	6
Total lymphocyte count (/mm^3^)	≥1600	1200–1599	800–1199	<800
Score	0	1	2	3
Total cholesterol (mg/dL)	≥180	140–179	100–139	<100
Score	0	1	2	3

**Table 2:** Demographic characteristics, surgical considerations, and comorbidities; data are presented as mean (CI95) or count (percentage). Abbreviations—BMI (body mass index); ENT (ear, nose, and throat surgery); DIEP (deep inferior epigastric artery perforator flap); ALT (anterolateral thigh flap).

Patient Group	Overall *n* = 72	No Complications *n* = 61	True Flap Loss *n* = 4	Any Flap Complications *n* = 11	*p*-Value
Demographical data					
Mean age, years	55.3 (51.5–59.1)	56.9 (61.0–65.4)	65.0 (63.5–66.5)	49.6 (37.7–56.1)	0.057
Sex (female), *n* (%)	32 (44.4%)	25 (40.1%)	2 (50.0%)	5 (45.5%)	0.418
Area of reconstruction					
Extremity, *n* (%)	15 (20.8%)	12 (19.6%)	-	3 (27.3%)	0.289
ENT, *n* (%)	26 (36.1%)	22 (36.1%)	2 (50.0%)	4 (36.4%)	0.496
Head and neck, *n* (%)	16 (22.2%)	14 (30.0%)	1 (25.0%)	2 (18.2%)	0.322
Breast, *n* (%)	15 (20.8%)	13 (21.3%)	1 (25.0%)	2 (18.2%)	0.457
Microvascular flap type					
ALT, (%)	32 (44.4%)	27 (44.3%)	2 (50.0%)	5 (45.5%)	0.828
Fibular flap, (%)	9 (12.5%)	8 (13.1%)	1 (25.0%)	1 (9.1%)	0.478
DIEP, *n* (%)	9 (12.5%)	7 (11.5%)	-	2 (18.2%)	0.528
Radial artery flap, *n* (%)	6 (8.3%)	6 (9.8%)	-	-	-
Other, *n* (%)	16 (22.2%)	13 (21.3%)	1 (25.0%)	3 (27.3%)	0.413
Indication for surgery					
Trauma, *n* (%)	8 (11.1%)	6 (10.1%)	-	1 (9.1%)	0.918
Oncology, *n* (%)	40 (55.6%)	32 (58.2%)	3 (75.0%)	6 (54.5%)	0.469
Defect, *n* (%)	19 (26.4%)	11 (20.0%)	1 (25.0%)	4 (36.4%)	0.511
Infection, *n* (%)	5 (6.9%)	5 (8.2%)	-	-	-
Comorbidities					
Coronary artery disease, *n* (%)	4 (5.6%)	3 (4.9%)	1 (25.0%)	1 (9.1%)	0.059
Diabetes mellitus, *n* (%)	5 (6.9%)	4 (6.6%)	-	1 (9.1%)	0.691
Hypertension, *n* (%)	28 (38.8%)	19 (31.1%)	3 (75.0%)	6 (54.5%)	0.133
Dyslipidemia, *n* (%)	16 (22.2%)	13 (21.3%)	1 (25.0%)	3 (27.3%)	0.624
Smoking history, *n* (%)	13 (18.1%)	11 (18.0%)	1 (25.0%)	2 (18.2%)	0.249
Obesity (BMI > 30 kg/m^2^), *n* (%)	12 (16.6%)	8 (13.1%)	2 (50.0%)	5 (45.5%)	0.010 **
Cerebrovascular accident, *n* (%)	4 (5.6%)	4 (6.6%)	-	-	0.620

The ** symbol is used to indicate statistical significance when comparing the group without complications to both the true flap loss group and the any flap complications group.

**Table 3:** Intraoperative and anesthesia considerations; data are presented as mean (CI95) or count (percentage).

Patient Group	Overall *n* = 72	No Complications *n* = 61	True Flap Loss *n* = 4	Any Flap Complications *n* = 11	*p*-Value
Duration of surgery, hours	6.39 (5.75–7.02)	6.33 (5.59–7.07)	7.63 (5.86–9.39)	6.66 (5.29–8.04)	0.235
Volume of intraoperative crystalloid, mL	2345.83 (2141.39–2550.28)	2352.50 (2133.31–2571.69)	2875.00 (1681.58–4068.42)	2312.50 (1608.14–3016.86)	0.145
Volume of intraoperative colloid, mL	506.25 (401.74–610.76)	482.50 (367.10–597.90)	500.00 (-)	625.00 (329.42–920.58)	0.471
Intraoperative colloid to crystalloid ratio	0.22 (0.17–0.27)	0.20 (0.15–0.25)	0.18 (0.10–0.27)	0.33 (0.09–0.56)	0.306
Intraoperative hematocrit, %	30.60 (29.20–32.00)	29.58 (27.70–31.45)	31.50 (25.15–37.85)	34.40 (30.32–38.48)	0.009 *
Use of vasopressors/sympathomimetics, *n* (%)	41 (56.90%)	36 (59.00%)	2 (50.00%)	6 (54.50%)	0.549

The * symbol is used to indicate statistical significance when comparing the group without complications to the any flap complications group.

**Table 4:** Biomarkers and nutritional systems for predicting any flap complications; data are presented as mean (CI95), median (IQR), or count (percentage).

Patient Group	Overall *n* = 72	No Complications *n* = 61	Any Flap Complications *n* = 11	*p*-Value
Biomarkers				
Lymphocyte count 10^9^/L	1.59 (1.39–1.79)	1.71 (1.49–1.92)	0.97 (0.67–1.26)	0.001 *
Monocyte count 10^9^/L	0.55 (0.48–0.62)	0.58 (0.51–0.66)	0.37 (0.22–0.51)	0.021 *
Lymphocyte/monocyte ratio	3.46 (2.91–4.02)	3.55 (2.90–4.20)	2.97 (2.28–3.65)	0.830
Mean plasma albumin, g/dL	3.94 (3.81–4.06)	3.96 (3.84–4.09)	3.79 (3.28–4.30)	0.631
Mean total plasma cholesterol, mg/dL	196.58 (185.21–207.95)	198.44 (186.43–210.45)	186.73 (147.93–225.53)	0.310
Nutritional assessment systems				
CONUT score	2(2)	2 (3)	3 (6)	0.013 *
CONUT ≤ 2	50 (69.4%)	46 (75.4%)	4 (36.4%)	0.009 *

The * symbol is used to indicate statistical significance when comparing the group without complications to the any flap complications group.

## Data Availability

The datasets used and analyzed during the current study are available from the corresponding author upon reasonable request. The corresponding author will ensure individual privacy is not compromised during the transfer of datasets.
